# Biodegradation of different PET variants from food containers by *Ideonella sakaiensis*

**DOI:** 10.1007/s00203-022-03306-w

**Published:** 2022-11-16

**Authors:** Andreas Walter, Laura Sopracolle, Mira Mutschlechner, Martin Spruck, Christoph Griesbeck

**Affiliations:** 1 Department of Biotechnology & Food Engineering, MCI—The Entrepreneurial School, 6020 Innsbruck, Austria; 2 Department of Environmental, Process & Energy Engineering, MCI—The Entrepreneurial School, 6020 Innsbruck, Austria

**Keywords:** *Ideonella sakaiensis*, Polyethylene terephthalate, qPCR, Waste management, Recycling, Micro-plastic

## Abstract

The accumulation of macro-, micro- and nano-plastic wastes in the environment is a major global concern, as these materials are resilient to degradation processes. However, microorganisms have evolved their own biological means to metabolize these petroleum-derived polymers, e.g., *Ideonella sakaiensis* has recently been found to be capable of utilizing polyethylene terephthalate (PET) as its sole carbon source. This study aims to prove its potential capacity to biodegrade two commercial PET materials, obtained from food packaging containers. Plastic pieces of different crystallinity were simultaneously introduced to *Ideonella sakaiensis* during a seven-week lasting investigation. Loss in weight, appearance of plastics, as well as growth of *Ideonella sakaiensis*—through quantitative real-time PCR—were determined. Both plastics were found enzymatically attacked in a two-stage degradation process, reaching biodegradation capacities of up to 96%. Interestingly, the transparent, high crystallinity PET was almost fully degraded first, followed by the colored low-crystallinity PET. Results of quantitative real-time PCR-based gene copy numbers were found in line with experimental results, thus underlining its potential of this method to be applied in future studies with *Ideonella sakaiensis*.

**Supplementary Information:**

The online version contains supplementary material available at 10.1007/s00203-022-03306-w.

## Introduction

Since its introduction in 1941, polyethylene terephthalate (PET) has become one of the most extensively applied polymers with a worldwide production of more than 400 million tons in 2020 (Skoczinski et al. 2021), predominantly used for the production of synthetic fibers and single-use plastic bottles and food containers (Wallace et al. 2020). Although PET is recyclable, high percentages of plastic waste accumulate in the ecosystems, causing a severe environmental burden, which is mostly undiscovered (Wallace et al. 2020; Prata et al. 2019; Chen et al. 2018). Hence, plastic degradation is one of the major global challenges, which have to be tackled urgently (Aer et al. 2022).

In 2016, a novel bacterium was detected, which may be advantageous to remove PET from the environment (Yoshida et al. 2016; Tanasupawat et al. 2016). Sampled in a PET recycling side in Sakai city, Japan, *Ideonella sakaiensis* (ISAK) is capable to utilize PET as its sole carbon source (Tanasupawat et al. 2016; Bornscheuer 2016). This Gram-negative strain reveals optimal growth rates at pH 7–7.5 and temperatures of 30–37 °C (Tanasupawat et al. 2016). Two key enzymes could be identified, both facilitating the degradation and assimilation of PET: the exo-enzyme PETase converts PET to mono-(2-hydroxyethyl) terephthalate (MHET) with a relatively high activity at room temperature, when compared to other PET-degrading enzymes (Aer et al. 2022). Then, in the cell periplasm, MHETase hydrolyzes MHET to the PET educts ethylene glycol (EG) and terephthalic acid (TPA), which is subsequently transported into the cytoplasm and introduced into the tricarboxylic acid cycle (Palm et al. 2019).

The aim of this study was to investigate the simultaneous biodegradation of two commercial PET-packing materials with different crystallinities—one colored PET (PETcol) and one transparent PET (PETtra), obtained from commercial food containers—by ISAK during an investigation period extending over 7 weeks in total. The proof of biodegradation capacities of commercial packaging materials, which were rarely investigated to date, will facilitate to understand the full potential of microorganisms and their enzymatic toolbox when it comes to the application of polymer recycling.

## Materials and methods

### Cultivation, media preparation and PET characterization

A pure culture of ISAK was obtained from the Biological Resource Centre, NITE (NBRC; Kazusakamatari, Japan) and cultivated in medium 802 (Wako Pure Chemical Industries, Ltd, Japan), according to NBRC online catalog. Growth temperature was set to 33.5 °C using a rotary shaker (150 rpm). Overnight cultures were cryo-conserved in 10% glycerine [v/v] at—80 °C to be used on demand in experiments.

Round PET pieces (Ø 6 mm), cut out from the sidewall of PETcol and PETtra with a hand-puncher were characterized (Table 1): all pieces were weighted on a MX5-Microbalance (Mettler Tole-do, Columbus, USA). Thickness was determined with an outside micro-meter (Digimatic Micrometer 0–25 mmm, Mitutoyo, Kawasaki, Japan). The crystallinity of both plastics was characterized by differential scanning calorimetry (DSC, Mettler DCS 12 E, Mettler Toledo, Switzerland). PET pieces (*n* = 3) were heated in a nitrogen atmosphere at a heating/cooling rate of 10 °C min^−1^. The enthalpies of the crystallization peak (Hc), and melt enthalpies (Hm) revealed values of 21.3 and 17.8 Jg^−1^ and 37.7 and 21.9 Jg^−1^, for PETtra and PETcol, respectively. The crystallinity (%) was calculated using the equation $\left( {{\text{Hm}} {-}{\text{Hc}}} \right)/{\text{Hm}}100) \times 100$ (Table 1).

**Table 1:** Characteristics of colored PET (PETcol) and transparent (PETtra)

	PETcol	PETtra
Origin	Cookie container*	Grape container*
Color	Brown	Transparent
Thickness (µm)	182 (± 0.47)	167 (± 2.29)
Weight (mg)	6.38 (± 0.46)	6.43 (± 0.58)
Crystallinity (%)	3.2 (± 1.93)	12.6 (± 1.35)

**Fig. 1:**
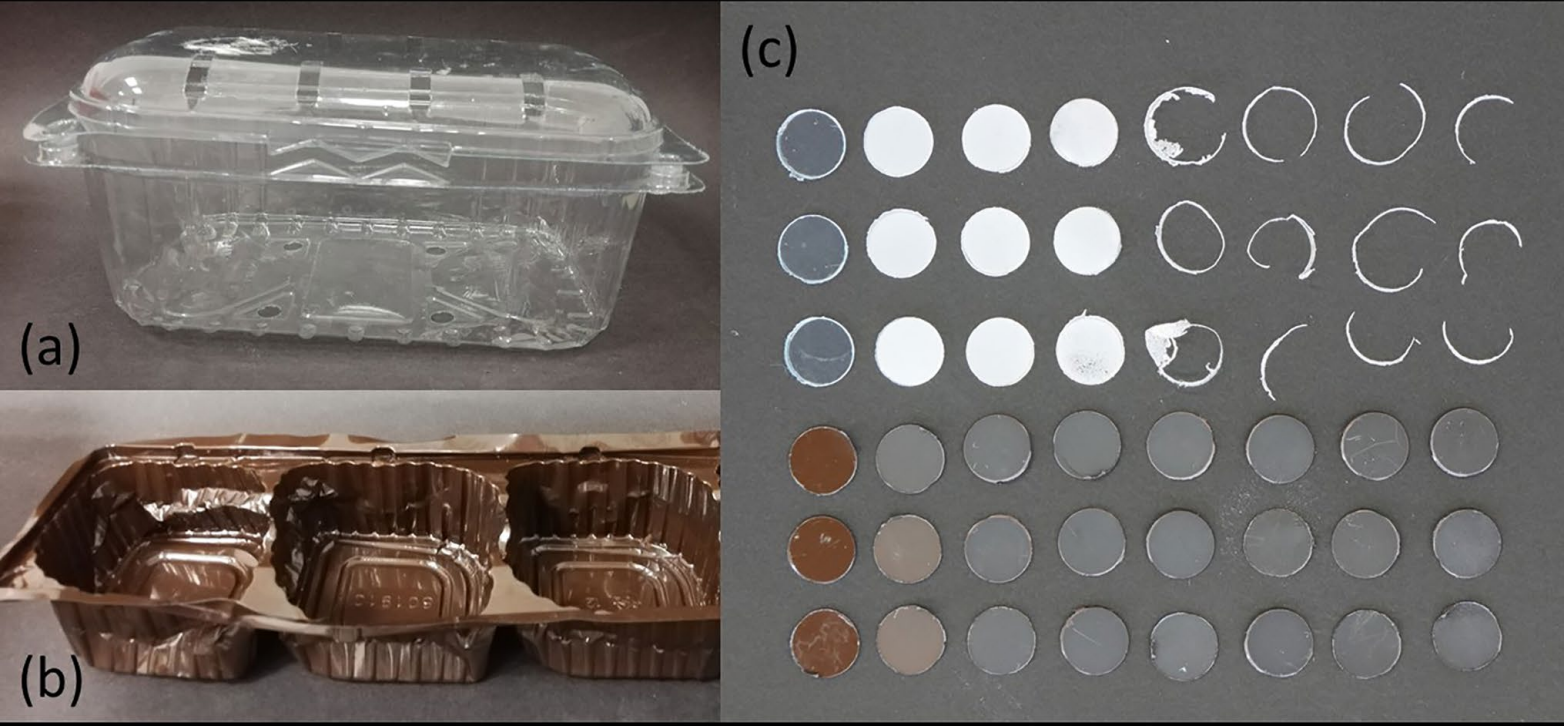
The two packaging materials **a** PETtra and **b** PETcol; **c** appearance, notified by eye, of PET negative control and enzymatically attacked PET after 1–7 weeks (from left to right)

*Pictures of containers are presented in Fig. 1.

### Degradation experiment

The degradation trials were conducted in shaking flasks containing 50 ml yeast extract–sodium carbonate–vitamins (YSV) medium, according to Tanasupawat et al. (2016). All PET pieces were sterilized in 70% ethanol for 48 h before application. Cell densities of 5.91 × 10^4^ (± 1.68 × 10^3^) CFU ml^−1^, obtained from ISAK cryo-cultures growing in medium 802 for 21.5 h, were used to inoculate treatments. Both PET materials were treated simultaneously in the same shaking flasks. Growth temperature was kept constant at 33.5 °C on a rotary shaker (150 rpm). Three shaking flasks were removed weekly to determine physicochemical and biological analyses while the rest of the flasks remained incubated. After 7 weeks, the last three treatments, as well as the negative controls (*n* = 3)—containing YSV and PET, without ISAK—and the positive controls (*n* = 3)—inoculated with ISAK in YSV, without PET—were removed to terminate the degradation experiment.

### Physicochemical parameters

PET pieces were removed through a sieve (0.5 mm mesh size; Karl Weis, Murr, Germany), carefully washed in ethanol (70%) and Aqua dest and dried at room temperature overnight prior to weight determination thereafter. Microscopic changes of PET pieces were documented, using a stereo microscope (Krüss, Hamburg, Germany) at a magnification of 15 × in combination with a Canon EOS 600D (Canon, Tokio, Japan). The pH was measured with a pH 3110 device (WTW, Weilheim, Germany) in the broth, immediately after PET pieces were removed.

To provide information on the surface microstructure, PET samples of weeks one, three, five and seven were analyzed using a JSM-IT200LA scanning electron microscope (SEM; JEOL, Tokyo, Japan) operated at 10 kV and supplied with a SE detector and a BSE detector. For sample preparation, PET pieces were sputtered with gold under an argon atmosphere, at a distance of 40 mm with a Sputter Coater 108auto (Cressington, Watford, UK).

### Quantitative real-time application

Samples were collected in triplicates after each week and stored frozen until further processing (the rest of the flasks remained incubated as stated before). The samples were filtered through a 0.45 µm membrane (Whatman, UK), washed and cut into pieces with a sterile scalpel prior to extraction. DNA was extracted using the NucleoSpin^®^ Microbial DNA Kit (Macherey–Nagel, Düren, Germany) according to the manufacturer’s instructions with proteinase K. Subsequently, the quantity and purity of the extracted DNA were evaluated via UV/VIS spectrophotometry with NanoDrop 2000cTM. Quantitative real-time PCR (qPCR) was performed on a CFX96 Touch Deep Well Real-Time PCR System (Bio–Rad, USA), with the primer pairs BAC338f and BAC805r (Yu et al. 2005) being applied. Prior to amplification, the samples were subjected to an initial denaturation step at 95 °C for 5 min. Each run included non-template controls (NTC, UltraPure DNase/RNase-Free Distilled Water, Invitrogen, USA) as well as DNA extracts from positive controls inoculated with ISAK but without any PET material and negative controls without inoculation (*n* = 3). After quantification, PCR products were checked via melting curve analysis. For construction of calibration curves, we used a genomic DNA standard from *Ideonella azotifigens* (DSM 21438) purchased from the German collection of microorganisms and cell cultures (DSMZ, Germany). Cycling conditions for quantification of the assays targeting bacterial 16S rRNA gene copies were as followed: 94 °C for 30 s, 60 °C for 30 s, and 72 °C for 90 s (35 cycles).

## Results and discussion

In this study, the potential of ISAK to biodegrade two PET-packing materials—one PETcol and one PETtra—obtained from commercial food containers was investigated in a 7-week lasing experiment. Appearance of PET, noticed by eye, changed within days, revealing opacity in PETtra and color change in PETcol (Fig. 1). Such observations were also noticed by Wallace et al. (2020), where amorphous PET, when treated with ISAK, became obviously cloudy within days. The PET surfaces became rougher and were found perforated after three and 7 weeks, for PETtra and PETcol, respectively. After 4 weeks, PETtra was found almost degraded, however revealing a residual ring of compressed PET (Suppl. Figure 1), due to cutting out PET pieces from plastic containers.

**Fig. 2:**
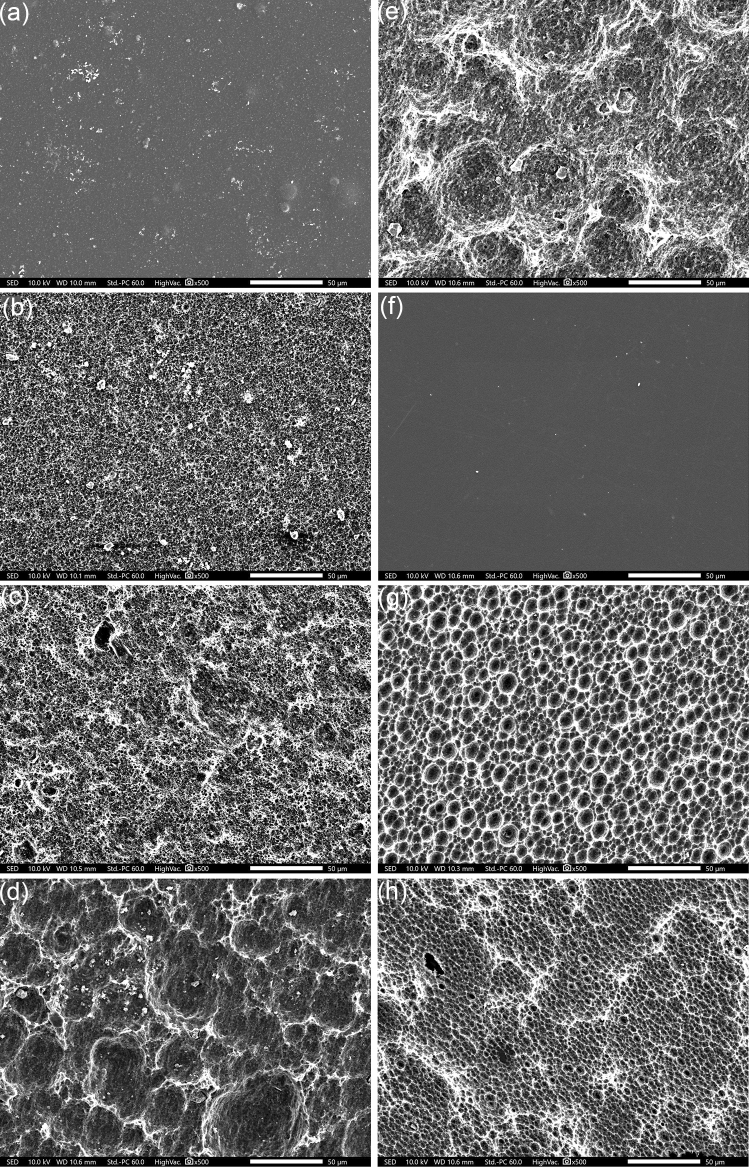
Biodegradation of initial PETcol (**a**) and PETtra (**f**) pieces under 500 × magnification over time: PETcol after one (**b**), three (**c**), five (**d**) and seven weeks (**e**), respectively. PETtra after one (**g**) and three (**h**) weeks, respectively. Thereafter, PETtra was found destructed, revealing a residual ring of compressed PET (Suppl. Figure 1). The white bars are indicating 50 µm

SEM observation (Fig. 2) confirmed enzymatic activity, causing extensive biodegradation on the smooth surface of initial PETcol (a) and PETtra (f). Pits and cavities were increasingly observed over time, revealing average diameters of 2.6 µm (± 0.71 µm), 3.44 µm (± 0.8 µm), 21.7 µm (± 9.99 µm) and 45.4 µm (± 9.08 µm) over time for PETcol (b–e), as well as 9.24 µm (± 3.19 µm) and 32.1 µm (± 4.39 µm) for PETtra, in the second and third week (g, h), respectively. Similar results in respect to plastic deconstruction were previously observed for gut microorganisms (Yang et al. 2014), hyper-thermophilic bacteria (Chen et al. 2020) and *Ideonella sakaiensis* (Yoshida et al. 2016).

The pH was found consistent throughout the overall experiment (7.03 ± 0.02), indicating suitable conditions for ISAK.

**Fig. 3:**
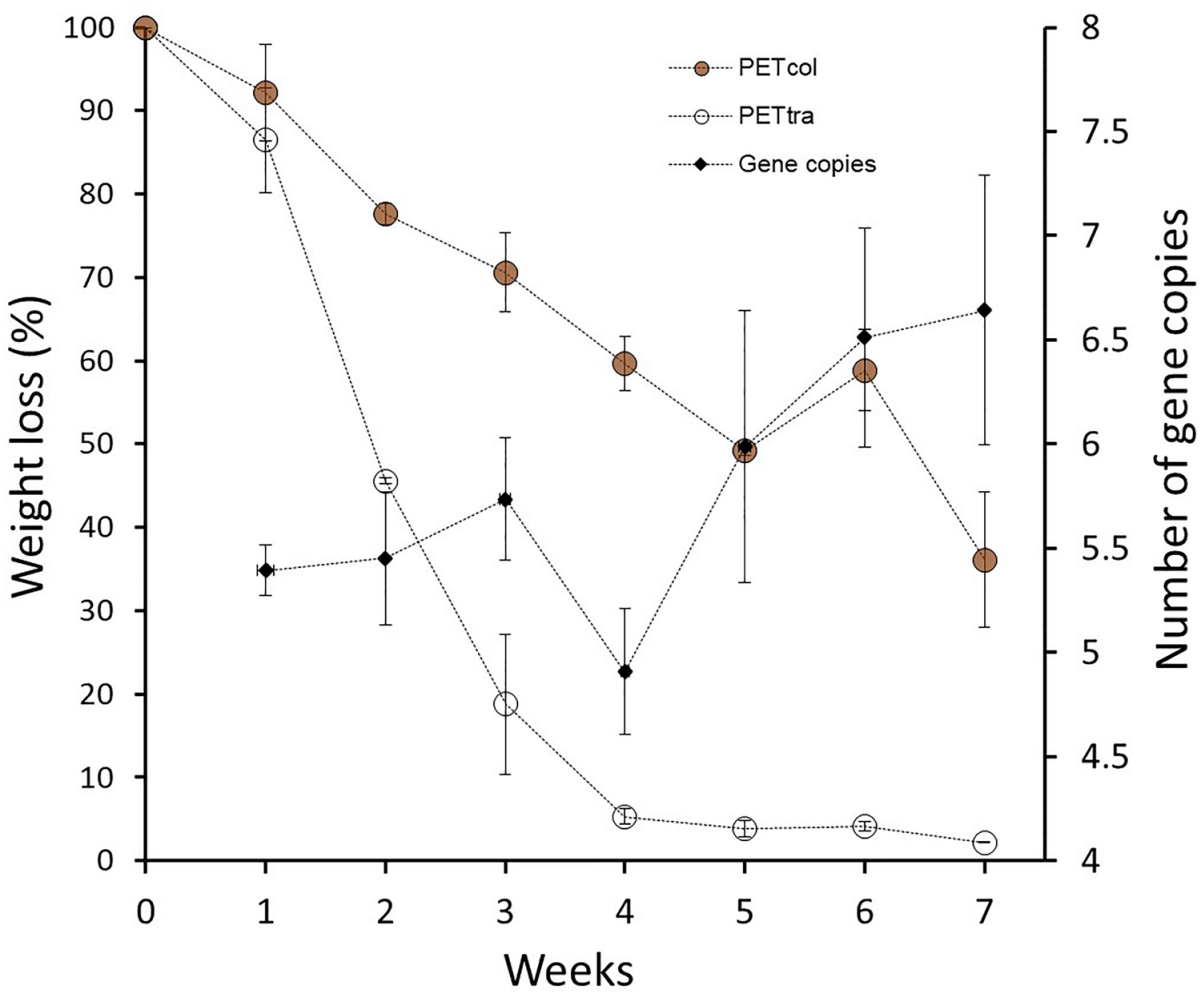
Degradation of the two PET-packing materials by *Ideonella sakaiensis* within 7 weeks. y-axes are representing weight loss of PET material (%, *n* = 3) and change in gene copy numbers (LOG10) of *Ideonella sakaiensis* (*n* = 3) during the 7-week lasting experiment. The positive controls inoculated with ISAK but without any PET materials revealed LOG10 abundances of 4.72 (± 0.17). No peaks were observed in the negative controls without inoculation as expected

Degradation capacities for PETtra were found strongly accelerated in comparison to PETcol, as shown in Fig. 3, revealing losses of 13.5% (± 6.27%) and 7.84% (± 6.13%) after 1 week, 54.4% (± 0.35%) and 22.4% (± 3.04%) after 2 weeks, and 81.2% (± 8.4%) and 29.4% (± 2.02) after 3 weeks for PETtra and PETcol, respectively. After 4 weeks, PETtra biodegradation almost stopped, revealing stagnating biodegradation capacities of 94.7% (± 0.93%), 96.2% (± 0.97%), 95.9% (± 0.55%) and 96.8% (± 0.18%) over the last 3 weeks. Biodegradation of PETcol remained gradually, revealing an endpoint capacity of 63.9% (± 17.5%). Negative controls, investigated after 7 weeks were found unaltered, revealing a weight loss of 0.05%.

qPCR results were found in line with degradation capacities. All approaches showed higher copy numbers when compared with the positive controls—containing ISAK but no PET—with LOG10 abundances of 4.72 (± 0.17). LOG10 abundances of 5.39 (± 0.12) were detected after 1 week of degradation, thereafter increasing slightly until week four, when PETtra was almost degraded revealing LOG10 abundances of 4.91 (± 0.3). Subsequently, copy numbers started to increase again, reaching 6.64 (± 0.65) after 7 weeks, probably due to PETcol degradation.

Although assimilation of PET films through ISAK was previously described in 2017, where Yoshida et al. found low crystallinity PET films (60 mg; 20 × 15 × 0.2 mm; 1.9% crystallinity) biodegraded by 33%, after 3 weeks of incubation in YSV broth at 30 °C (Yoshida et al. 2016), degradation capacities of commercial packaging materials were rarely investigated to date. In 2020, Wallace et al. investigated the degradation of different parts of a commercial PET bottles and reported that the bacterium was not capable to grow from high crystalline PET, which is the majority of plastic in PET bottles, within a 2-week lasting experiment (Wallace et al. 2020). On the contrary, anamorph or low crystalline parts, such as finish, shoulder top and the base of the bottle, as well as solidified anamorph PET, formerly of higher crystallinity, could be degraded efficiently under the same conditions. Authors estimated that percentages of non-degradable parts of plastic bottles could range from 52 to 82%. PET bottles as well as polyester textiles usually reveal crystallinities of 30–40% when compared to other PET packaging materials, ranging from approximately 6–8% crystallinity (Kawai et al. 2014; Ronkvist et al. 2009). Interestingly, in this research, crystallinity was not affecting degradation efficiency overall. Although ISAK was found capable to tackle both materials, with lower-crystalline PETcol showing accelerated biodegradation, when introduced simultaneously with PETtra. Authors have already proposed other important factors, such as (i) hydrophobicity, (ii) surface topography, or (iii) molecular size of synthetic polymers are important factors affecting their biodegradability (Tokiwa and Calabia 2007; Webb et al. 2012; Wei and Zimmermann 2017). However, for this study, it remains unclear which PET characteristics effected enzymatic degradation in particular.

## Conclusion

In this study, we investigated the biodegradation potential of two commercial PET materials through *Ideonella sakaiensis*. This bacterium was capable to almost degrade all PET from a grape container within 4 weeks and to reduce PET, obtained from a cookie container, by more than 50% within 7 weeks. It could be shown that both plastics were simultaneously exploited, however revealing different degradation rates, as expected. A qPCR using universal bacterial primers was shown to be in line with the physicochemical results obtained and this molecular–biological approach may be used in future studies as well to reveal DNA quantities of plastic-degrading microorganisms. While predicted natural lifetimes of PET range from 25 (Liu et al. 2019) to hundreds of years (Austin et al. 2018), biotechnological approaches, under sufficient conditions, could open novel grounds in industrial application, e.g., a complete biodegradation to face pollution or the recycling of the monomers TPA and/or EG (Taniguchi et al. 2019).

Big potentials bear the technologies of genetic engineering: (Genetic Engineered Ideonella Sakaiensis Bacteria: A Solution of the Legendary Plastic Waste Problem 2018) proposed to modify ISAK genes with *Azotobacter *sp. genes to boost its survival capability in habitats, such as soil and water. Authors have demonstrated that PETase could be successfully expressed in *Chlamydomonas reinhardtii* and *Escherichia coli*, respectively (Aer et al. 2022; Kim et al. 2020; Shi et al. 2021). Others (e.g., (Ma et al. 2018; Son et al. 2019; Meng et al. 2021)) have screened PETase mutants and variants to improve enzymatic performance as well as thermal stability. In addition, screenings for other, potent plastic-degrading microorganisms have to be continued, including (1) thermophilic microorganisms for industrial applications and (2) salt-tolerant microorganisms to face ocean pollution (Taniguchi et al. 2019). As proposed by Karunatillaka et al. (2020), there are a significant number of previously uncharacterized proteins that hold the capability of plastic biodegradation. Beside degradation, these enzymes hold potentials for bio-sensing applications of pollutants (Gul et al. 2021).

## Supplementary Information

Below is the link to the electronic supplementary material.

Supplementary file1 (JPG 325 KB)
