# Supplementary figures and images for: Biodegradation of different PET variants from food containers by Ideonella sakaiensis
Source: Arch Microbiol. 2022 Nov 16;204(12):711. doi: 10.1007/s00203-022-03306-w (PMC9668955; doi:10.1007/s00203-022-03306-w)

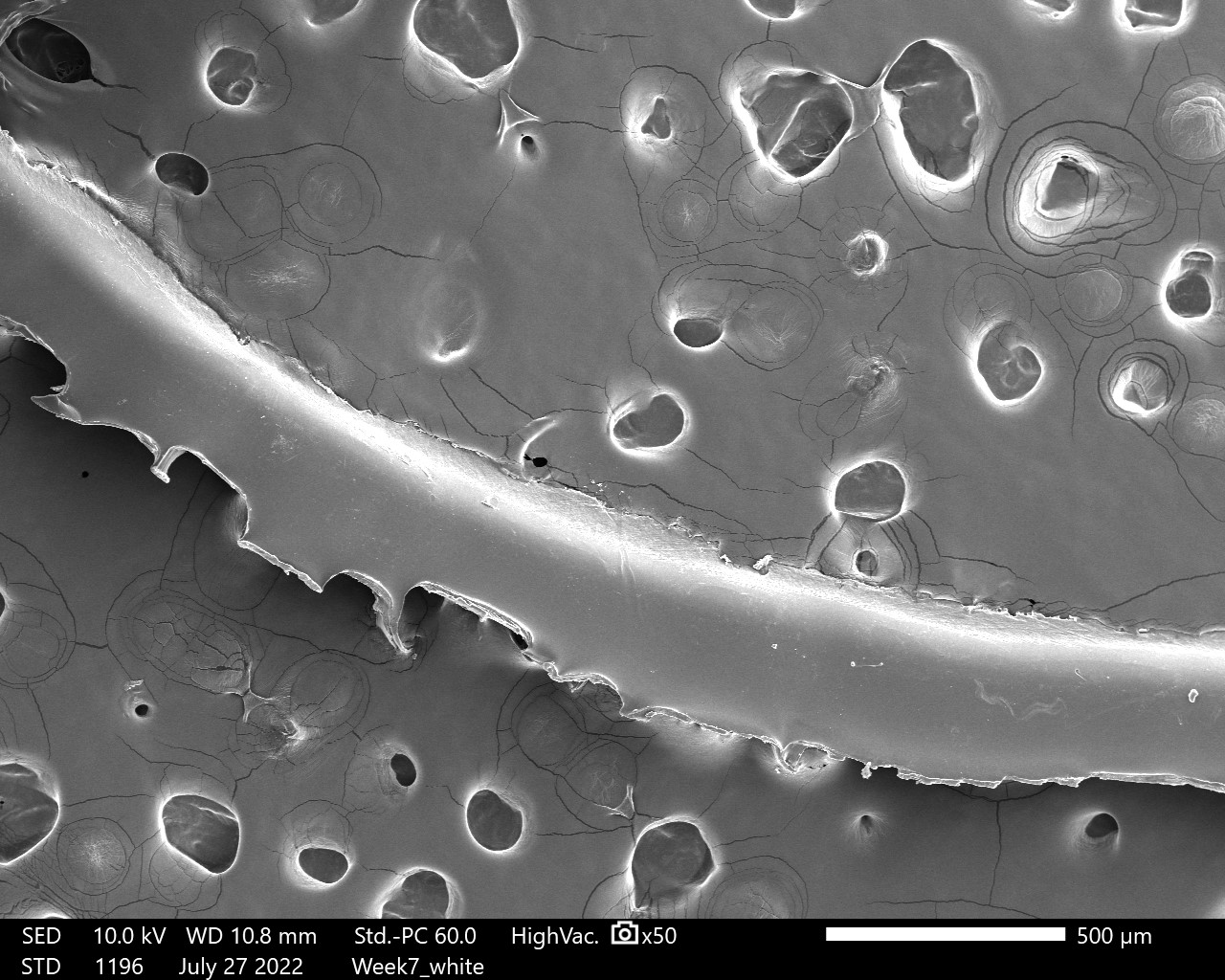

Supplement: Supplementary file 1 — Supplementary file1 (JPG 325 KB) [file 203_2022_3306_MOESM1_ESM.jpg]
